# Early Diagnosis of Atrial Fibrillation and Stroke Incidence in Primary Care: Translating Measurements into Actions—A Retrospective Cohort Study

**DOI:** 10.3390/biomedicines11041116

**Published:** 2023-04-07

**Authors:** Josep-Lluis Clua-Espuny, Pedro Molto-Balado, Jorgina Lucas-Noll, Anna Panisello-Tafalla, Eulalia Muria-Subirats, Josep Clua-Queralt, Lluïsa Queralt-Tomas, Silvia Reverté-Villarroya, Investigators EBRICTUS Research

**Affiliations:** 1 Primary Health-Care Centre, Institut Català de la Salut, Primary Care Service (SAP), EAP Tortosa-Est, Plaça Carrilet s/núm, 43500 Tortosa, Spain; 2 Research Support Unit Terres de l’Ebre, Institut Universitarid’Investigació en Atenció Primària Jordi Gol (IDIAP JGol), USR Terres de l’Ebre, 43500 Tortosa, Spain; 3 Primary Health-Care Centre, Institut Català de la Salut, Primary Care Service (SAP) Terres de l’Ebre, UUDDTortosa-Terres de l’Ebre, 43500 Tortosa, Spain; 4 Health Department, Management CatSalut Terres de l’Ebre, 43500 Tortosa, Spain; 5 Primary Health-Care Centre, Institut Català de la Salut, Primary Care Service (SAP) Terres de l’Ebre, EAP Amposta, C/Sebastià Juan Arbó, 139, 43870 Amposta, Spain; 6 Primary Health-Care Centre, Institut Català de la Salut, Primary Care Service (SAP), EAP Tortosa-Oest, Avda Cristobal Colon, 16, 43500 Tortosa, Spain; 7 Nursing Department, Campus Terres de l’Ebre, University Rovira i Virgili, Av Remolins, 13, 43500 Tortosa, Spain; 8 Advanced Nursing Research Group, Medicine and Health Sciences, University Rovira i Virgili, 43002 Tarragona, Spain

**Keywords:** atrial fibrillation, stroke, neurocognitive impairment, risk management, screening

## Abstract

(1) Background: AF-related strokes will triple by 2060, are associated with an increased risk of cognitive decline, and alone or in combination, will be one of the main health and economic burdens on the European population. The main goal of this paper is to describe the incidence of new AF associated with stroke, cognitive decline and mortality among people at high risk for AF. (2) Methods: Multicenter, observational, retrospective, community-based studies were conducted from 1 January 2015 to 31 December 2021. The setting was primary care centers. A total of 40,297 people aged ≥65 years without previous AF or stroke were stratified by AFrisk at 5 years. The main measurements were the overall incidence density/1000 person-years (CI95%) of AF and stroke, prevalence of cognitive decline, and Kaplan–Meier curve. (3) Results: In total, 46.4% women, 77.65 ± 8.46 years old on average showed anAF incidence of 9.9/10^3^/year (CI95% 9.5–10.3), associated with a four-fold higher risk of stroke (CI95% 3.4–4.7), cognitive impairment(OR 1.34 (CI95% 1.1–1.5)), and all-cause mortality (OR 1.14 (CI95% 1.0–1.2)), but there was no significant difference in ischemic heart disease, chronic kidney disease, or peripheral arteriopathy. Unknown AF was diagnosed in 9.4% and of these patients, 21.1% were diagnosed with new stroke. (4) Conclusions: The patients at high AF risk (Q4th) already had an increased cardiovascular risk before they were diagnosed with AF.

## 1. Introduction

Atrial fibrillation (AF) and stroke are two common conditions that disproportionately affect the elderly population. AF is expected to rise by 2.5 times in the next 50 years, is often asymptomatic, and has a recognized association with other comorbidities, which contribute to the increased risk of stroke and adverse events. A 34% increase in strokes related to AF is predicted in the coming decades, the number of ischemic strokes recorded in people above 80 years of age will triple (2010–2060), and there will be an estimated 27% increase in stroke survivors who develop these diseases, alone or in combination. Therefore, AF will become one of the main health and economic burdens on the European population [1,2,3,4,5,6,7]. Both AF and stroke incidence rates will increase with age, and the aging of the global population has contributed to a rise in the prevalence of these conditions with multifactorial and complex causes. Age-related changes in the heart, such as fibrosis and decreased conductivity, can increase the risk of developing AF as well as other factors such as hypertension, diabetes, obesity, and a history of heart disease or heart failure. Similarly, stroke risk factors include hypertension, diabetes, atrial fibrillation, smoking and atherosclerosis.

Stroke is already one of the leading causes of death and long-term disability in developed countries and the second highest burden of disease in Europe due to its social and economic impacts [5,8]. One out of four strokes is recurrent, and secondary stroke carries a greater risk than first stroke of death and disability. A link was reported between silent cerebral infarction detected by MRI and atrial myopathy with an increased risk of developing cognitive impairment, dementia [9] and a range of different cardiovascular diseases referred to as major adverse cardiovascular events (MACEs) [10,11]. Ischemic stroke in people with AF [12,13,14] is characterized by greater severity and disability, increasing costs by up to 20%, and its incidence is 2.3-fold higher among people ≥75 yearsold. Moreover, up to 50% will suffer from residual disability, insufficient cognitive ability and/or poor mental health [15,16].

Given the prevalence of AF and the fact that its complications will increasein the coming decadesbecause of the aging population, itisaprioritytodevelop proposals aimed at improving diagnosis and treatment. Among these approaches, strategies for the opportunistic detection of AF are recommended by international organizations including the European Society of Cardiology (ESC), Stroke Alliance for Europe (SAFE), European Heart Rhythm Association (EHRA), Royal College of Physicians of Edinburg (RCPE), World Healthcare Forum (WHF), European Primary Care Cardiovascular Society (EPCCS), and Health Information and Quality Authority (HIQA). However, there is disagreement about whether opportunistic screening detects AF more effectively than the usual practice [17,18,19].

The Action Plan in Europe (2018–2030) [6] prioritizes the availability of detection and treatment programs in primary care to improve the diagnosis and monitoring of populations at risk of AF in the respective health contexts of each country, a lack of information prevails on whether an elevated risk exist for the correlation between AF and stroke before the diagnosis of AF. As a result, it remains paramount to identify patients at elevated risk of AF to determine who would benefit from risk factor control and treatment. Conventional practices involve the use of clinical risk scoring criteria to identify patients at risk, but these risk scores have modest discriminatory power. The past decade has seen substantial advances in the diagnostic and treatment options available to minimize the impact of acute ischemic stroke, new insights have been gained on the utility of biomarkers and imaging modalities, and there are emerging data on the importance of the identification of subclinical AF using wearable devices in primary care practice. The main goal of this study is to describe the incidence of new cases of AF associated with the diagnosis of stroke as well with cognitive decline and major adverse cardiovascular events among people at high risk of AF.

## 2. Materials and Methods

### 2.1. Study Design

This was an observational, retrospective, multicenter, and community-based study of a cohort of 40,297 of the general population aged 65 to 95 years between 1 January 2015 and 31 December 2021 without a prior diagnosis of atrial fibrillation or stroke. The protocol received ethics evaluation and approval from the Ethical Committee of Jordi Gol University Institute of Primary Care Research with registration number P15/047.

### 2.2. Study Scope

The study was conducted in the primary care setting of Terres de l’Ebre (Catalonia, Spain) (Appendix A). According to census data, the territory comprises 178,112 inhabitants (women, 49.6%), with a higher aging index (159.5) than Catalonia (131.3) and Spain (118.4) [20]. This is relevant to the demographics of the study because most of the cohort was made up of older individuals [21,22].

The public health service is made up of four counties with a total of 11 primary care teams (EAPs), all managed by the Catalan Health Institute, Department of Health (CatSalut). In total, 98.2% of the census population has an active clinical record in at least one of the EAPs and/or reference hospitals of the territory. This availability of digitalized clinical history allows for continuous follow-up care from any center.

### 2.3. Data Collection and Information Sources

The clinical background data were obtained retrospectively from a computerized database, provided to the principal investigator by the Information and Communication Technology Department from the minimum basic dataset at hospital discharge (CMBD-HA) register using the specific International Classification of Diseases (10th version; ICD-10) in a fully encrypted format. The particular datasets utilized for this project were as follows:

1. The “Health Plan of the *“Terres de l’Ebre”* region 2021–2025 [23]: a digital access platform used by the Department of Health.
2. The Institute of Statistics of Catalonia for each region of the territory: demographics, inhabitant density/km^2^, and aging index vs. Catalonia (100%).
3. The HCC3 Patient Episode Dataset for Catalonia (CatSalut, Health Department), which includes demographic and clinical data on all daily inpatient and outpatient admissions in Catalonian hospitals.
4. The 11 EAPs (Catalonian Health Institute, Governmental agency) share a clinical information database for all general practice (E-cap, HC3) and hospital (E-sap) interactions, including clinical data, symptoms, investigations, diagnoses, comorbidities, prescribed medications, referrals to secondary and tertiary care, and status (alive/dead). Pharmacological variables were collected from the SIRE (Catalan acronym for Integrated Electronic Prescription System).

Data on these factors were collected automatically when possible, or manually otherwise.

### 2.4. Study Population

Initially, the study included people 65–95 years-old, resulting in a total of 55,459 patients. After applying inclusion criteria, 40,297 people without AF were included in the study. All the patients enrolled were followed up for the occurrence of atrial fibrillation after the inclusion. In this analysis, the primary outpoint was the outcome of ischemic stroke. Other secondary outcomes investigated cognitive impairment, cardiovascular outcomes (MACEs), and all-cause death. The hypothesis was that the incidence of stroke, cognitive impairment, and all-cause mortality would be higher in individuals at high risk for AF before its diagnosis.

### 2.5. Inclusion and Exclusion Criteria

#### 2.5.1. Inclusion Criteria

Patients 65–95 years old with a high risk of AF [24], active medical records in any of the health centers with information accessible through the shared history (HCC3), without prior AF or stroke, residence in the territory, and assignment to any of the territory’s primary are teams (EAP). The non-availability or loss of accessibility to the information necessary for the study was considered as a reason for exclusion.

#### 2.5.2. Exclusion Criteria

Previous diagnosis of AF and/or stroke, non-availability of AF-index prognosis [25], pacemaker or defibrillator wearer, absence of or lack of access to individual or theirclinical records for any reason, difficulty in following the instructions, patient’s non acceptance of conditions, and/or residence outside the Terres de l’Ebre.

### 2.6. Variables

The information on AF and co-morbidities relevant to cardiovascular risk were obtained until loss-to-follow-up, date of death, or 31 December 2021, whichever occurred first. Atrial fibrillation was diagnosed according to the guidelines of the European Society of Cardiology. All new AF diagnoses were verified by two research physicians blind to the MEANS diagnosis. A cardiologist was consulted when consensus was not reached. Patients were classified according to the presence of AF. In cases of AF diagnosed during the follow-up period, data were extracted at the time of AF diagnosis or until the end of follow-up. Data for patients who did not present AF during follow-up were obtained according to the mean during follow-up:

(1) Cardiovascular risk factors and diagnostics using specific International Classification of Diseases (ICD–10) code prefixes for cerebrovascular disease (ischemic stroke or transient ischemic attack, I63, G45), heart failure (I50-51), ischemic heart disease (stable or unstable angina, percutaneous coronary intervention, coronary artery bypass grafting or myocardial infarction) (I20-I25), hypertension (I10–I15), hypercholesterolemia (E78), diabetes mellitus (E10–E14), body mass index (BMI), chronic kidney disease (CKD) (N18) and estimated glomerular filtration rate (eGFR ml/min/1.73 m^2^).
(2) Clinical scores: AF risk index, CHADsVASc score, Pfeiffer Short Mental Status Questionnaire (SPMSQ) score, NIHSS score, and modified Rankin scale (mRS) in case of stroke as recommended by current guidelines. The model to stratify the risk of suffering AF at five years among individuals aged ≥65 years was published previously [24,25].
(3) Antiplatelet and/oral anticoagulation (antivitaminK vs. NOACs).
(4) Vital status (dead/alive) at the end of the study. All participants were followed from 1 January 2015 to 31 December 2021, loss tofollow-up, or date of death, whichever occurred first.

According to the guidelines of the European Society of Cardiology, the performance of screening for AF registered in the electronic medical records (e-cap) of any citizen aged ≥65 yearswho contacted the health system during the study period was evaluated. Eventually, 359 randomized patients at high risk of AF received a wearable Holter device (Nuubo^TM^) for 4 weeks. Expert cardiologists evaluated the anonymized Holter records to identify AF episodes. Full details on protocol and results of the AFRICAT study have been previously reported elsewhere [26] (AFRICAT: Atrial Fibrillation Research in CATalonia, NCT03188484).

### 2.7. Statistical Analysis

The characteristics of the population were defined through a descriptive statistical analysis. Baseline characteristics are presented as counts and percentages, mean and standard deviation (SD) for normally distributed continuous variables, or median and interquartile range (IQR) for non-normally distributed continuous variables, as appropriate. Quantitative variables were examined with Student’s *t-*distribution for independent samples while qualitative variables were analyzed with the chi-square distribution according to bivariate analysis for normal distributions.

The mathematical formula of the model was applied to the target population, and the quartiles of the distribution from lowest to highest risk were defined (Q1st–Q4th), with the Q4th (high risk) being of interest. The AF incidence density/1000 people/year (ID), the incidence of MACEs, and the registered prevalence of cognitive decline were calculated for each group. The incidence rate was calculated in person-years, and the denominator was the sum of the length of time for which each person was observed, totaled for all persons. This denominator represents the total time the population was at risk of and being monitored for disease. The odds ratio (OR) risk increase for each vascular outcome associated with atrial fibrillation was calculated by the event in the exposure group divided by the odds of the event in the control or non-exposure group. Absolute risk increases were expressed in events per 1000 people/year of follow-up. Kaplan–Meier curves were used for mortality assessment, to compare survival probabilities, and to identify any significant differences. Two-sided *p-*value <0.05 was considered statistically significant. All statistical analyses were conducted using IBM SPSS Statistics version 21.0.

## 3. Results

### 3.1. Baseline Characteristics

The patient’s baseline characteristics according to study groups are shown in Table 1. In total, 40,297 people without a personal history of AF were included. The average age of the patients was 77.88 ± 8.47 years, 46.48% were women, and the follow-up time was 80.65 ± 9.5 months. There were significant differences between the AF patterns for all the risk factors of interest at baseline. In total, 18.15% died during follow-up. Those with AF were significantly older (81.22 ± 7.91 vs. 77.65 ± 8.46 years, *p* < 0.001), and the most prevalent cardiovascular risk factors were arterial hypertension (HTA) (75.5%), dyslipidemia (47.6%), and diabetes (29.8%).

### 3.2. Atrial Fibrillation Incidence

**Figure 1:**
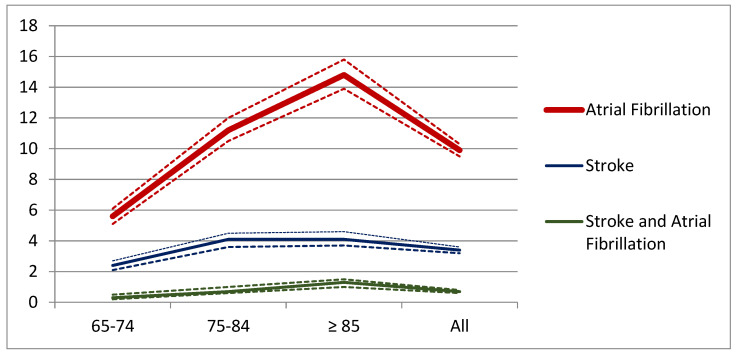
Atrial fibrillation and stroke incidence density rates for every 1000 people per year (CI95%), by age group.

The AF incidence (ID) was 9.9/1000 people per year (CI95% 9.5–10.3) and increased in line with the AFrisk levels, reaching 17/1000 people-years (CI95% 16.1–18.1) among those at the highest risk of AF (Figure 1), with a significantly higher incidence among men (OR 1.23 (CI95% 1.14–1.33)) than women. However, in the fourth quartile, the incidence of AF was higher among women (OR 1.52 (CI95% 1.35–1.71)). The average age (81.22 ± 7.91) was significantly higher than that of the overall group.

### 3.3. Atrial Fibrillation and Stroke Incidence

In total, 885 stroke episodes were confirmed among the patients with a high risk of AF. The diagnosis of AF was found to be associated with a four-fold higher risk of stroke (OR 4.03 (CI95% 3.43–4.74)), and the highest stroke incidence was 11.1/1000 people/year (CI95% 9.6–12.8). The stroke incidence displayed a significant linear correlation with the Rankin scores (*p* < 0.001) and Pfeiffer scores (*p* < 0.001). In total, 187 of the strokes (21.12%) were associated with atrial fibrillation (Table 2). The factors associated with AF and strokes were different from those associated with atrial fibrillation alone. These factors included higher CHA_2_DS_2_-VASc and Charlson scores, higher MACE’s incidence, higher rates of cognitive impairment, and higher rates of mortality than those without stroke.

Most strokes (78.8%) occurred in people without AF, but 65.7% occurred in the Q4thrisk level, especially among women (88.17%). The stroke incidence increased in line with the AFrisk levels, reaching 6.8/1000 people-years (CI95% 6.2–7.5), and with the mean value (*p* < 0.001) on the Rankin scale. The incidence rate of stroke with AF was significantly higher (11.1/10^3^-year, (CI95% 9.6–12.8, *p* < 0.001)) than among those without AF (2.7/10^3^-year (CI95% 2.5–3.0, *p* < 0.001)). In total, 57.1% of patients with a simultaneous diagnosis of stroke and AF were in the Q4th group and displayed higher NIHSS scores (7.25 ± 8.62 vs. 4.55 ± 5.74, *p* = 0.002).

Screening for AF was reported in 74.5% of the population ≥65 years old. From the sample of 359 individuals, new AF was diagnosed in 34 subjects (9.47%) during the Holter monitoring period. Up to 82.35% of the cases of AF were recorded during the first 7 days and up to 88.23% during the first two weeks. The number of patients screened that was required to detect one new AF case in the study was 15. Unknown AF was diagnosed in 9.47% of people at high risk of AF, among whom 21.1% were diagnosed with new stroke.

### 3.4. Atrial Fibrillation and Cognitive Impairment

The 41.7% of cases with cognitive impairment were concentrated in the Q4th risk level, were older (84.84 ± 6.70 vs. 81.22 ± 7.91, *p* < 0.001), and already had a higher incidence of cognitive impairment and mortality before their diagnosis of AF.

The risk of cognitive impairment (OR 1.34 (CI95% 1.19–1.51, *p* < 0.001) was higher not only with a new diagnosis of AF, but also with the association between AF and stroke (*p* = 0.001). There was a progressive increase in prevalence (2.6% up to 15.3%), and there was an association of cognitive deterioration with AFrisk level as well as a significant linear correlation between AFrisk score and Rankin score (0.66 ± 1.15 vs. 2.27 ± 1.53, *p* < 0.001) and Pfeiffer score ((2.13 ± 3.06 vs. 3.86 ± 3.42, *p* < 0.001), but not with the NIHSS score (*p* = 0.150) after a stroke episode.

### 3.5. Atrial Fibrillation and Cardiovascular Comorbidities

Those with a new AF (Table 3) had a significantly high incidence of cardiovascular comorbidities and all-cause mortality. However, individuals in the Q4th quartile of AF risk had similar incidence rates of ischemic heart disease, chronic kidney disease, and peripheral arteriopathy prior to their AF diagnosis, as compared to those who were newly diagnosed with AF.

## 4. Discussion

In this large study of people at high AF risk, we present the results related to its incidence, unknown prevalence, and association with a higher risk of heart failure, ischemic heart disease, and stroke; along with the prevalence of cognitive impairment and all-cause mortality. Atrial fibrillation is considered a chronic and progressive disorder [7,10,27,28]. Cardiovascular risk is already higher before AF diagnosis, especially in the case of chronic kidney disease, ischemic heart disease and peripheral arterial disease. Moreover, aging is often associated with comorbidities, polypharmacy, and frailty, which can further increase the risk of AF and stroke, and patients who develop stroke while on antiplatelet therapy have a higher likelihood of developing atrial fibrillation after stroke [29]. The interplay between these factors is not yet fully understood, and further research is needed to better identify the causes. Nonetheless, it is necessary to translate all these measurements into early meaningful actions such as an early diagnosis, structured management, and optimization of cardiovascular risk factors and comorbidities to approaches in improving outcomes of AF patients.

Ninety percent of strokes are related to modifiable risk factors, and despite progress in the diagnosis and management of AF, the modification of these risk factors remains the cornerstone [7]. Several studies have noted that AF, HTA and diabetes mellitus are highly prevalent, frequently undiagnosed, and not optimally treated despite their high risk of stroke [4,6,7,30]. Furthermore, across Europe, primary and secondary prevention strategies do not appear to work well enough to control the major risk factors, and the proportion of people with a history of stroke with unhealthy lifestyle factors is increasing [31]. In addition, the results show an increasing incidence of AF and stroke from 65 years of age and onward, and the largest gap between the prevalence of AF and the estimated incidence occurred between 65 and 74 years of age, with a rate of undiagnosed AF of 2.2% (CI95% 1.3–3.1) [32].

Theuse ofpulse palpationin an ordinary visit has been recommended as the first step in screening to detect AF, but this has a lower sensitivity than other methodologies using devices [33,34,35], as screening studies have found a prevalence of unknown atrial fibrillation in 10–66% in patients with risk factors. There was no difference between systematic and opportunistic screening [36], and furthermore, screening did not reduce stroke incidence [37,38], although organization of the screening process can be more significant than the technical solutions used for assessing heart rhythm. This suggests that the success of screening programs may be influenced by various factors, such as the traits of the target population [39], the accessibility of resources, technology utilized, the involvement of healthcare professionals, and the level of community engagement and education [18].

Previously unknown AF was diagnosed in 9.4% of the monitored sample of individuals at high risk of AF, and a lower number of screened patients required to diagnose one case of AF was shown (NNS = 15) compared with the NNS of 147 required using the opportunistic detection method [26]. Furthermore, in primary care, the use of health technologiesfor heart rhythm monitoring may improve the detection of AF, especially among people at high risk.

Although the clinical benefits of early anticoagulation are widely recognized and safe, 26.9% did not achieve appropriate control objectives [18,38,39,40,41,42,43]. This fact increases the impact of the unknown AF (Table S1). The rate of ischemic stroke in patients with AF ≥ 75 years old was 2.3 times higher and was associated with greater severity and disability as well as a 20% increase in stroke-related costs [13,14]. For this reason, different international associations [41] have recently proposed extra cost associated with poor control and/or the non-use of oral anticoagulants to the total cost of stroke care. About one-third of the annual treatment cost of a patient with AF can be attributed to anticoagulation management [43,44]. With regard to other prevalent risk factors such as hypertension and diabetes, they are disproportionally affected by the risk of major outcomes, but only 40% of patients who have had a stroke episode are correctly treated. Given the frequent association with diabetes and hypertension in the populations with AF [43,45] and its use among the risk factors to stratify thromboembolic risk in AF patients, the importance of active AF screening among hypertensive and diabetic individuals is highlighted [24,25,46].

Despite the wide variability in the estimated degree of stroke preventability from the perspective of risk factor control [47,48], at least 1082 strokes/year (8.3–14.2% of all the strokes/year in Catalonia) associated with previously undiagnosed AF could be avoided (Table S1). Therefore, 5662 cases of unknown AF may be diagnosed through the device-based monitoring of people at high risk of AF. According to stroke costs [2], the estimated potential savings could be around EUR 260 million/year in a short time horizon, without including the costsaving associated with the prevention of long-term disability and the saving of lives. In addition to the epidemiological estimate associated with demographic aging, the greater cardiovascular comorbidity, frequency, average drug consumption, mortality and severity of stroke confirm the estimated increase in the costs associated with the treatment of stroke episodes associated with AF. The accuracy of screening is crucial, but the health outcomes resulting from screening compared to no screening have not been evaluated. Potential harms of screening includethe misinterpretation of records, which can lead to false reassurance or false alarms as well as to the possible initiation of unnecessary treatment or known risks of appropriate treatment [49].

The prevalence of cognitive disorder is higher in each quartile in relation to that in the general population [50]. Several studies [51,52,53,54] have reported echocardiographic criteria and several biomarkers as prognostic factors for the development of dementia and new AF, raising the possibility of a new approach to early detection. However, how this may affect prevalence seems to be unknown. The presence of a progressive increase in the prevalence and severity of cognitive impairment with the risk of AF would support a possible etiopathogenic interrelation between both processes in the general population [55], as well as the need to protocolize its detection [56].

A model of comprehensive care for AF showed a 45% reduction in mortality from any cause [27,28], but its analysis was subsequent to the diagnosis of AF. A pathway referred to as “Atrial fibrillation Better Care” (ABC) has been proposed to streamline a more holistic or integrated care approach to atrial fibrillation (AF) management and has been associated with a reduced risk of major adverse events, including mortality, thromboembolism, and MACE [27,54]. In the territory of study [23], cardiovascular diseases are the main causes of death, and stroke is the main etiology related to years of lives lost and disability among women. The results highlight that the highest incidence of AF, stroke, cognitive impairment, and mortality were concentrated among women in the fourth quartile. This may reflect inequality in health, and women in the fourth quartile should be a priority for AFscreening in primary care. In total, 23% of patients who have suffered a stroke will suffer a second stroke, with higher rates of disability (36% to 51%) and increased mortality (20% to 34%) [4,6,7,16]. Secondary prevention measures have the potential to reduce the number of stroke survivors who suffer additional strokes by 80%. International best practice guidelines recommend a multifaceted approach to secondary stroke prevention and care [56,57,58] addressing both technological support for timely medical decisions and the effective provision of self-management tools and recommendations for stroke survivors and their careers.

Currently, biomarkers [26,59], electronic devices [60,61,62,63], and machine learning techniques [64,65] are new tools in AF screening and may improve its effectiveness [66]. The application of a clinical risk model (Figure S1) could optimize the selection of candidates for screening even further, and early anticoagulation and early treatments such as cryoablation or drug therapy [67] may modify the chronic progression of atrial fibrillation, lowering stroke risk rates. Around 80% of the participants diagnosed with AF in the United Kingdom population are eligible for early cardiac rhythm control [68], and the implementation of new digitalhealth technologieshas the potential to improve outcomes by facilitating self-management and by enabling earlier detection and intervention for adverse events [69,70,71]. Finally, the use of artificial intelligence (AI) approaches in stroke risk prediction showed a significant ability to predict the risk of stroke occurrence, but it did not significantly improve discriminative accuracy for new-onset stroke compared with pooled cohort equations [72].

As potential limitations of the study, the authors consider the following: the under-registration of diagnoses; the cross-sectional format used does not allow for the definition of causal relationships between AF, silent stroke and cognitive impairment even in the absence of stroke, and the fact that the results are limited to a generic AF and cognitive decline and do not account for differences in ages, type of AF and cognitive dysfunction. It must be considered that the estimates of mild cognitive impairment in general population studies include all cases, regardless of their likelihood of being detected in the health care system or the underlying disease etiology. The strengths of the study include the considerable number of cases, the long follow-up, and the fact that the study was conducted in the general population [24,25] using a validated statistical model that predicts the probability of suffering from AF based on their covariates prior to the inclusion of patients in order to reduce a possible bias. The target population for screening is yet to be established, particularly with regard to the impact of oral anticoagulation on cognitive outcomes. At present, ideal strategies for screening for AF remain to be defined. Future research will aim to focus on the interrelation of high-riskAF models, silent stroke, cognitive impairment and the cost-effectiveness analysis of a protocol that should include the systematic identification of patients along the AF risk scale, AF burden, type of cognitive disorder, modification of risk factors, use of echocardiographic and imaging criteria, biomarkers, and technological support tools, including electronic devicesand machine learning techniques. This may resolve the uncertainties related to the most effective type of monitoring and the question of whether to start anticoagulant treatment as well as supporting the definition of therapeutic strategies to prevent AF-related cognitive decline.

## 5. Conclusions

1. The individuals with the higher riskAF (Q4th) already had a similar risk to those with AF for ischemic heart disease, chronic kidney disease, or peripheral arteriopathy before their diagnosis of AF.
2. Unknown AF was diagnosed in 9.47% of patients at high risk of AF (Q4th) and among 21.1% of those with a new stroke. The NNS to detect one new case of AF was 15.
3. Individuals with prevalent AF had higher incidence of cardiovascular disease (MACE), four-fold higher risk of stroke, cognitive impairment (OR 1.34 (CI95% 1.1–1.5), and all-cause mortality (OR 1.14 (CI95% 1.0–1.2).
4. Stroke incidence increased progressively with AF risk levels. The 57.1% of simultaneous diagnoses of stroke and AF occurred in the Q4th risk level. The cardiovascular profile of individuals with AF and stroke was found to be different from those with atrial fibrillation alone.
5. The 41.7% of cases with cognitive impairment were concentrated in the Q4th risk level, were older (84.84 ± 6.70 vs. 81.22 ± 7.91, *p* < 0.001), and already had a higher incidence of cognitive impairment and mortality before diagnosing AF and displayed higher NIHSS (7.25 ± 8.62 vs. 4.55 ± 5.74, *p* = 0.002) scores than those without AF.

## 6. Patents

Patent AFRICAT: Diagnosis markers for atrial fibrillation (EP19382321.8).

## Figures and Tables

**Table 1:** Baseline characteristics: no atrial fibrillation vs. newly diagnosed atrial fibrillation.

Variables	All	No AF	AF	*p*
All (*n*)	40,297	37,723	2574	
Female	18,878	17,535	1343	<0.001
Age average	77.88 ± 8.47	77.65 ± 8.46	81.22 ± 7.91	<0.001
Arterial hypertension	25,555	23,610	1945	<0.001
Diabetes mellitus	10,458	9689	769	<0.001
BMI (kg/m^2^)	28.71 ± 5.16	28.66 ± 5.14	29.5 ± 5.38	<0.001
Dyslipidemia	19,129	17,913	1216	0.822
Active Smoking	838	809	29	0.854
Risky Alcohol	506	487	19	0.395
Ischemic cardiomyopathy	2915	2558	357	<0.001
Heart failure	2772	2096	676	<0.001
Stroke	885	698	187	<0.001
Peripheral vascular disease	2776	2431	345	<0.001
Dementia/cognitive impairment	3781	3471	310	<0.001
Antiplatelet therapy	6251	6110	141	<0,001
CHA_2_DS_2_-VASc	3.24 ± 1.16	3.20 ± 1.15	3.83 ± 1.2	<0.001
Anticoagulation	2981	987	1994	<0.001
AntivitaminK	1698	754	944	<0.001
NACO	1288	235	1053	<0.001
Death—all causes	7317	6799	518	0.008

**Table 2:** Characteristics of people with newly diagnosed AF with stroke and without stroke.

Variables	All	AF and Stroke	AF without Stroke	*p*
All (N)	2574	187	2387	<0.001
Women (*n*)	1343	112	1231	0.565
Men (*n*)	1231	97	1134	
Average age (years ± SD)	81.95 ± 8.46	82.91	81.87	0.899
Arterial hypertension (*n*)	2035	152	1883	0.667
Diabetes mellitus (*n*)	788	57	731	0.935
BMI (kg/m^2^ average ± SD)	28.71 ± 5.16	28.62 ± 5.21	29.40 ± 5.37	0.052
Glomerular filtration rate (mL/min/1.73 m^2^)	72.17 ± 18.89	65.92 ± 20.13	62.84 ± 20.43	0.069
Dyslipidemia (*n*)	1263	86	1177	0.374
Ischemic cardiomyopathy (*n*)	370	24	346	0.591
Heart failure	716	50	666	0.738
Peripheral vascular disease(*n*)	360	26	334	1.000
MACE (*n*)	1162	209	953	<0.001
CHA_2_DS_2_-VASc (average ± SD)	3.83 ± 1.19	4.76 ± 1.1	3.74 ± 1.17	<0.001
Anticoagulation (*n*)	2026	134	1892	0.016
Pfeiffer score (average ± SD)	3.02 ± 3.07	3.38 ± 2.91	2.98 ± 3.09	0.332
Dementia/cognitive impairment (*n*)	342	41	301	<0.001
Charlson score (average ± SD)	1.81 ± 1.43	2.60 ± 1.36	1.75 ± 1.42	<0.001
Statins treatment (*n*)	957	91	866	=0.001
Death—allcauses (*n*)	2574	137	2437	0.008

**Table 3:** Odds ratio of atrial fibrillation vs. no-atrial fibrillation vs. high AF risk (4th quartile).

	High AF Risk (Q4th)	New AF	No AF	OR AF/Q4 (CI95%)	OR AF/NoAF (CI95%)
N	10,072	2574	37,718		
AF all Incidence/1000 people per year (CI95%)	1148 17.3 (16.3–18.3)	2574 9.9 (9.5–10.3)	-		
Women *n* (%) Incidence/1000 people per year (CI95%)	2876 22.8 (20.7–25.1)	1231 8.9 (8.4–9.4)	-		
Men *n* (%) Incidence/1000 people per year (CI95%)	7196 15.0 (13.9–16.1)	1343 11.0 (10.4–11.6)	-		
Stroke/Transient ischemic attack Incidence/1000 people per year (CI95%)	456 6.9 (6.2–7.5)	187 3.4 (3.2–3.6)	698 2.7 (2.5–3.0)	1.62 (1.37–1.92) *p* < 0.001	4.03 (3.43–4.74) *p* < 0.001
Heart Failure Incidence/1000 people per year (CI95%)	1.844 27.5 (26.3–28.8)	676 40.1 (37.1–43.2)	2.096 8.3 (7.9–8.6)	1.45 (1.33–1.6) *p* < 0.001	4.85 (4.5–5.3) *p* < 0.001
Ischemic Heart Disease Incidence/1000 people per year (CI95%)	1468 22.0 (20.8–23.1)	367 21.8 (19.6–24.1)	2558 10.1 (9.7–10.5)	0.99 (0.88–1.11) *p* = 0.908	2.16 (1.93–2.41) *p* < 0.001
Major Cardiovascular Events Incidence/1000 people per year (CI95%)	3791 56.3 (54.5–58.1)	1230 73.0 (68.9–77.1)	5352 21.1 (20.5–21.6)	1.29 (1.21–1.38) *p* < 0.001	3.52 (3.31–3.75) *p* < 0.001
Cognitive Impairment Incidence/1000 people per year (CI95%)	1553 23.3 (22.1–24.5)	310 18.4 (16.4–20.6)	3471 13.7 (13.2–14.1)	0.78 (0.69–0.89) *p* = 0.002	1.34 (1.19–1.51) *p* < 0.001
Chronic Kidney Disease Incidence/1000 people per year (CI95%)	2731 40.8 (39.3–42.3)	676 40.1 (37.1–43.2)	5158 20.3 (19.8–20.9)	0.98 (0.90–1.06) *p* = 0.706	1.97 (1.82–2.13) *p* < 0.001
Peripheral Arteriopathy Incidence/1000 people per year (CI95%)	1.337 20.0 (18.9–21.1)	345 20.5 (18.4–22.7)	2.431 9.6 (9.2–10.0)	1.02 (0.90–1.15) *p* = 0,724	2.13 (1.90–2.4) *p* < 0,001
Death—allcauses Incidence/1000 people per year (CI95%)	2822 42.5 (40.9–44.0)	518 30.7 (28.1–33.5)	6799 26.8 (26.1–27.4)	0.72 (0.65–0.79) *p* < 0.001	1.14 (1.04–1.25) *p* = 0.027

**Figure A1:**
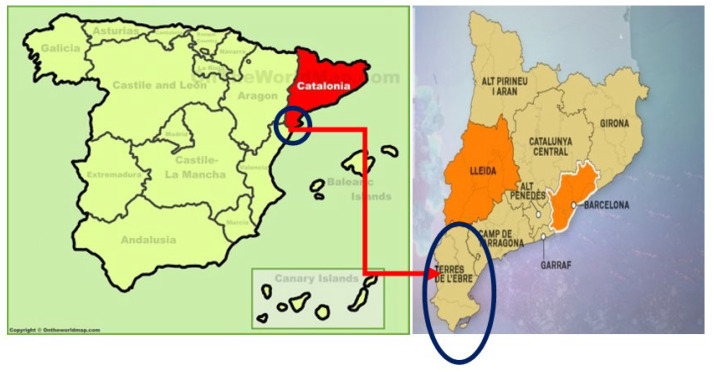
The application of a clinical risk model.

## Data Availability

Datasets were deposited in a publicly available database (https://github.com/Hipocrates57/Atrial_fibrillation/blob/main/Base%2065_95_quartils_DATASET.sav, accessed on 14 February 2023) or (https://github.com/Hipocrates57/Atrial_fibrillation.git, accessed on 14 February 2023).

## Supplementary Materials

The following supporting information can be downloaded at: https://www.mdpi.com/article/10.3390/biomedicines11041116/s1, Table S1: Estimated avoidable strokes by early diagnosis of atrial fibrillation (census Catalonia2020 [21]).
