# Supplementary material for: Early Diagnosis of Atrial Fibrillation and Stroke Incidence in Primary Care: Translating Measurements into Actions—A Retrospective Cohort Study
Source: Biomedicines. 2023 Apr 7;11(4):1116. doi: 10.3390/biomedicines11041116 (PMC10135492; doi:10.3390/biomedicines11041116)
Supplement: Supplementary file 1 [file biomedicines-11-01116-s001.zip › biomedicines-2259563-supplementary.pdf]

Table 1S. Estimated avoidable strokes by early diagnosis of Atrial fibrillation (census Catalonia 2020 [21])

People ≥ 65 year-old [21]	1,758,232	Estimated N
A/ Overall estimated Atrial Fibrillation Prevalence [33]	10.9% (CI95% 9.1-12.8)	191,647
B/ Registered AF prevalence in clinical records.	7.5%	131,867
C/ Unknown estimated Atrial Fibrillation prevalence (%) [33]	31.1%	59,793
D/ No-treatment with oral anticoagulant (%) [33]	26.9% (CI95%, 22.7–30.9)	35,472
Unknown AF prevalence (C)<br><br>Known prevalence without anticoagulant treatment (D)	N= 59,793 (C)<br><br>N = 35,472 (D)	[C+D] = 95,265
(E) Unknown AF diagnosis by Holter monitoring (%)	9.47%	5,662<br><br>[C-E] = 54,130
Adjusted Stroke rate (100-year) to CHADsVASc average (3.83±1.19)	4-9.8% year	2,165-5,304
(F) Avoidable strokes-year [39-41]	On average, blood thinners reduce the risk of an AF-related stroke by more than 50%.	1,082-1,856

The region of Catalonia includes a total population of 7.5 million inhabitants and approximately 13.000 stroke patients get admitted in the Catalan hospitals every year. All stroke code activations and reperfusion therapies in Catalonia are recorded in the Code Ictus Catalunya (CICAT) registry—a government-mandated, prospective, hospital-based data set [23].
